# Autologous nasal chondrocytes delivered by injectable hydrogel for in vivo articular cartilage regeneration

**DOI:** 10.1007/s10561-017-9649-y

**Published:** 2017-08-16

**Authors:** Wenliang Chen, Changhua Li, Maoxiu Peng, Bingju Xie, Lei Zhang, Xiaojun Tang

**Affiliations:** Department of Orthopedics, The Third Affiliated Hospital of Wenzhou Medical University, 168th Ruifeng avenue, Rui’an, 325200 People’s Republic of China

**Keywords:** Nasal chondrocyte, Cartilage repair, Alginate, Hydrogel, Tissue engineering

## Abstract

Cell based tissue engineering serves as a promising strategy for articular cartilage repair, which remains a challenge both for researchers and clinicians. The aim of this research was to assess the potential of autologous nasal chondrocytes (NCs) combined with alginate hydrogel as injectable constructs for rabbit articular cartilage repair. Autologous nasal chondrocytes were isolated from rabbit nasal septum, expanded either on monolayer or in 3D alginate hydrogel. In vitro, DNA quantification revealed that NCs can proliferate stable in 3D alginate matrix, but slower than that cultured in monolayer. Further, a higher synthesis rate of glycosaminoglycans (GAGs) was detected by GAG measurement in 3D alginate culture. Gene expression analysis at different time point (day 1, 7, 14) showed that 3D culture of NCs in alginate up-regulated chondrogenic markers (Col2A1, ACAN SOX9), meanwhile down-regulated dedifferentiation related gene (Col1A1). In vivo, autologous nasal chondrocytes combined with alginate hydrogel were used for repairing rabbit knee osteochondral defect (Alg + NC group). Histological staining indicated that Alg + NC group obtained superior and more hyaline-like repaired tissue both at 3 and 6 months after surgery. Mechanical analysis showed that the repaired tissue in the Alg + NC group possessed similar mechanical properties to the native cartilage. In conclusion, nasal chondrocytes appeared to be a very promising seed cell source for cartilage tissue engineering, and alginate hydrogel can serve as suitable delivery system.

## Introduction

Duo to trauma, inflammation, and degeneration, articular cartilage injuries are very common in trauma surgery as well as orthopaedics. Unfortunately, owing to its hypocellularity and avascular structure, the natural healing and regeneration ability of cartilage is very low and inefficient (Huey et al. 2012). Without early and effective treatment, cartilage injuries usually progress soon in joint damage, dysfunction and eventually lead to severe osteoarthritis. Several methods have been developed to promote cartilage repair. One of the most frequently used treatments in clinic is known as microfracture, which works by osteochondral drilling to recruit mesenchymal stem cells (MSCs) from bone marrow to cartilage injury site. However, clinic follow up showed that microfracture usually lead to the formation fibrocartilage which is apparently inferior to the native hyaline cartilage function (Bouwmeester et al. 2002; Matsunaga et al. 2007). Autologous chondrocyte implantation (ACI) has been applied successfully to promote hyaline cartilage regeneration, nevertheless, there are drawbacks need to be solved (Ebert et al. 2015). The autologous chondrocytes are mostly harvested from native cartilage within nonweight bearing areas of the joint, which may result in donor site morbidity (Matricali et al. 2010). In addition, chondrocytes tend to lose their chondrogenic phenotype with the increase of passage in 2D monolayer expansion, which will finally lead to inferior cartilage repair (Darling and Athanasiou 2005; Schrobback et al. 2011).

Cartilage tissue engineering which combines seed cells, scaffolds, and growth factors serves as a promising alternative strategy to support cartilage repair and regeneration. Nevertheless, some issues still remained to be addressed for purpose of achieving functionally engineered cartilage (Yang et al. 2017). The seed cell source is one of the most important elements in cartilage tissue engineering. Clinically, articular chondrocytes (ACs) are one of the most reported seed cell sources. However, the difficulties of harvesting sufficient cell amount and donor site morbidity have become bottlenecks of its extensive application (Matricali et al. 2010; Yin et al. 2016). Embryonic stem cells, induced pluripotent stem cells (iPSCs), and MSCs (such as bone marrow derived MSCs and adipose derived MSCs) have been intensively studied as alternative cell sources. Whereas, obstacles such as in vivo tumorigenesis, spontaneous differentiation, ectopic osteogenesis and adipogenesis have to be overcame (Zhang et al. 2013). NCs isolated from nasal septum have been reported as promising alternative cell source for cartilage tissue engineering for the following advantages: (1) NCs exhibit greater chondrogenic potential and higher capacity to generate hyaline cartilage like tissue than ACs (Rotter et al. 2002; Tay et al. 2004); (2) NCs could response similarly to ACs to biomechanical stimulation resembling joint loading (Candrian et al. 2008); (3) NCs and ACs are both derived from hyaline cartilage tissue with similar ECM components (Pelttari et al. 2014); (4) NCs are easy to harvest from a small nasal septum biopsy, with minimal donor site morbidity (Pelttari et al. 2017). Biological scaffold, which can provide structural support, mimic ECM microenvironment, and even influence cellular differentiation, is another important component of tissue engineering. Injectable hydrogels have been extensively studied in tissue engineering designs. Among which, naturally derived alginate, presenting a chemical structure similar to glycosaminoglycans which are the main components of cartilage ECM has drawed lots of researchers’ attention (Stilhano et al. 2016). Alginate hydrogel can provide a three-dimensional environment that support ACs proliferation and even maintain their chondrogenic phenotype, and can promote chondrogenic differentiation of MSCs. What’s more, alginate can serve as injectable vehicle for cell delivery with minimal invasion. Thus, alginate hydrogel showed great potential in cartilage repair and regeneration (Chang et al. 2009).

The aim of this study was to investigate whether NCs have potential for articular cartilage repair and regeneration. And we proposed alginate hydrogel as injectable cell delivery system of NCs for in vivo transplantation. We first cultured NCs in 3D alginate hydrogel. The results showed that NCs can proliferate stable in alginate and demonstrated superior GAG deposition compared with culture in monolayer. Moreover, 3D alginate culture of NCs showed to maintain its chondrogenic phenotype with the increase of culture time in vitro, which was revealed by relative gene expression analysis. Further, the isolated autologous NCs were delivered by alginate into cartilage defect for in vivo cartilage repair and led to superior hyaline-like articular cartilage repair in a rabbit model.

## Materials and methods

### Isolation of nasal chondrocytes

With ethical approval from the Institutional Animal Care and Use Committee of The Third Affiliated Hospital of Wenzhou Medical University, autologous rabbit NCs were isolated from the nasal septum biopsy (2 mm diameter) of adult New Zealand white rabbit (3.5–4.0 kg) under anaesthesia with 3% sodium pentobarbital (40 mg/kg body weight, i.p.). Briefly, the cartilage specimen was cut into very small slices and digested at 37 °C with 0.15% protease (Sigma, St-Louis, USA) in Hank’s balanced sodium salt (Invitrogen, Carlsbad, CA) for 30 min. Slices were then digested overnight at 37 °C with 0.025% type II collagenase in DMEM (Invitrogen, Carlsbad, CA) on a magnetic stirrer. Finally, the isolated NCs were cultured in DMEM supplemented with 10% FBS, 100 U/mL penicillin, 100 µl g/mL streptomycin, and 1% l-glutamine (Invitrogen, Carlsbad, CA) (culture medium). The NCs were maintained at 37 °C in a humidified atmosphere of 5% CO _2_, 20% oxygen and the culture medium was changed every 2–3 days.

### Three-dimensional culture of nasal chondrocytes in alginate hydrogel

P3 NCs were suspended at a density of 1 × 10^6^ cells/mL in 1.2% (w/v) low viscosity alginate (Sigma, Poole, UK) solution. 102 mmol/L CaCl_2_ was then added to form solid calcium alginate hydrogel beads. The beads were cultured in 6-well plate and the culture medium was changed every 2–3 days. Monolayer culture of NCs with the same cell amount also conducted as control.

### DNA and sulfated GAG quantification

Quantitative analysis of GAG and DNA content in monolayer and alginate hydrogel beads were performed at different time point (day 1, 3, 7, 14). Samples were digested with papain cocktail (125 mg/mL papain, 5 mM EDTA, 5 mM l-cysteine, and 100 mM phosphate buffer, pH 6.5) at 60 °C overnight. 1,9-dimethylmethylene blue dye assay was subsequently used for the measurement of total GAG (Yin et al. 2016). The total DNA content was determined by a PicoGreen DNA kit (Invitrogen) according to the manufacturer’s protocol and a Hiatachi F-700 Fluorescence Spectrophotometer was used for the measurement.

### RNA isolation and quantitative polymerase chain reaction (qPCR)

RNA isolation and qPCR of monolayer and alginate hydrogel beads were conducted at different time point (day 1, 7, 14). Total RNA was extracted from cells cultured in monolayer or alginate beads using Trizol reagent (Invitrogen) according to the manufacturer’s protocol. AMV First-Strand cDNA Synthesis kit (Invitrogen) was used for cDNA synthesis. For quantitative PCR, the RT^2^ SYBR Green Fluor qPCR Master mix (Qiagen) was used. The quantitative PCR was performed on an iCycler iQ™ Real-Time PCR Detection System (BioRad) with a setting of denaturation at 95 °C for 10 min followed by 40 cycles of 30 s at 95 °C, 60 °C for 1 min and 72 °C for 30 s. The qPCR results are reported as fold change in gene expression relative to the p0 NCs. The primers designed for qPCR are as follows: Col2A1 (forward 5′-CACGCTCAAGTCCCTCAACAAC-3′ and reverse 5′-TATCCAGTAGTCACCGCTCTTCC-3′), Col1A1 (forward 5′-AGCCCAGCATTGCCCAGAAG-3′ and reverse 5′-GCTCTCGCCGAACCACACG-3′), Aggrecan (forward 5′-TTCCCTGGCGTGAGAACCTAC-3′ and reverse 5′-CCTCCATCTCCTCTGCGAAGC-3′), SOX-9 (forward 5′-CGTGGTGACAAGGGTGAGAC-3′ and reverse 5′-TAGGTGATGTTCTGGGA GGC-3′), GAPDH (forward 5′-CCACTTTGTGAAGCTCATTTCCT-3′ and reverse 5′-TCGTCCTCCTCTGGTGCTCT-3′).

### Rabbit articular cartilage defect repaired with autologous nasal chondrocytes

Twenty four adult New Zealand white rabbits weighing 3.5–4.0 kg were used according to protocols approved by the institutional Animal Care and Use Committee at The Third Affiliated Hospital of Wenzhou Medical University. Autologous NCs were isolated from each rabbit as described before, and then expanded in monolayer to get sufficient cell amount for in vivo transplantation. The expanded P2 NCs were suspended at a density of 1 × 10^6^ cells/mL in 1.2% low viscosity alginate solution, and then transplanted into cartilage defect of the same rabbit.

Animals were anesthetized with 3% sodium pentobarbital (40 mg/kg body weight, i.p.), and then a cylindrical osteochondral defect of 3 mm in diameter and 2 mm in depth was created with a sterile biopsy punch in the trochlear groove center of both knee joint. Rabbits were randomly averagely divided into three groups according to the implants: the Alg + NC group, in which alginate containing NCs was injected into the osteochondral defects, and 103 mM CaCl_2_ was added to enable in situ gelation; The Alg group, in which the osteochondral defects was treated with alginate hydrogel without cells; The Defect group, in which the osteochondral defects was left empty. The rabbits were sacrificed by CO_2_ asphyxiation at 3 or 6 months after surgery.

### Histological analysis and scoring

Repaired knee (4 samples for each group) were fixed in 4% paraformaldehyde for 48 h at room temperature, decalcified for 4–5 weeks in 10% (w/v) EDTA in PBS at 37 °C. The decalcified samples dehydrated in a graded ethanol series, embedded in paraffin, and cut into 8 μm thick serial sections. Hematoxylin and eosin (H&E) staining and safranin O staining were performed for morphological evaluation and glycosaminoglycan content analysis of repaired tissue. For histological scoring, the histological sections of each group (n = 10) from repaired areas were blindly scored by three experienced evaluators based on the Wakitani scoring system (Wakitani et al. 1994).

### Micro-CT analysis

To evaluate the subchondral bone regeneration level within the defects, a small animal CT scanner (eXplore Locus Ultra, GE Healthcare, USA) was used to scan the femoral condyle samples. Rabbit knee samples were fixed in 4% paraformaldehyde for 48 h at room temperature, and then were loaded on a sample holder with the femur axis perpendicular to the scanning plane. The samples were scanned through a 360° rotation angle with a rotation interval of 1° with an exposure setting of 80 kV, 450 µA. The pixel size was 20 µm. Microview software and eXplore Reconstruction Utility software (GE Healthcare) were used to reconstruct and analyse the image data. A cylindrical region of interest (ROI) 3 mm in diameter within the repaired area was selected. Bone volume fraction (BVF), bone mineral density (BMD), tissue mineralized density (TMD), trabecular bone thickness (Tb.Th), spacing (Tb.Sp), and number of trabecular bone (Tb.N) were quantified.

### Biomechanical analysis

Nanoindentation testing was conducted to analyse the biomechanical properties of repaired tissue (n = 5) at 6 month after surgery using the TriboIndenter (Hysitron Inc, Minnesota, USA) with a 100-mm radius of a curvature conospherical diamond probe tip (Dai et al. 2014). Indentations were force-controlled to a maximum indentation depth of 500 nm. The samples were firstly loaded for 10 s, then hold for 2 s at the maximum depth, and unloaded for 10 s at last (Huang et al. 2014).

### Statistical analysis

SPSS 11.0 (SPSS, Chicago, IL) was used for statistical analyses. All date are expressed as mean ± SD. The differences between groups were evaluated using one-way analysis of variance with a post hoc test, and *p* < 0.05 was considered to indicate statistical significance.

## Results

### Proliferation and cartilage matrix production of NCs in 3D alginate culture

**Fig. 1:**
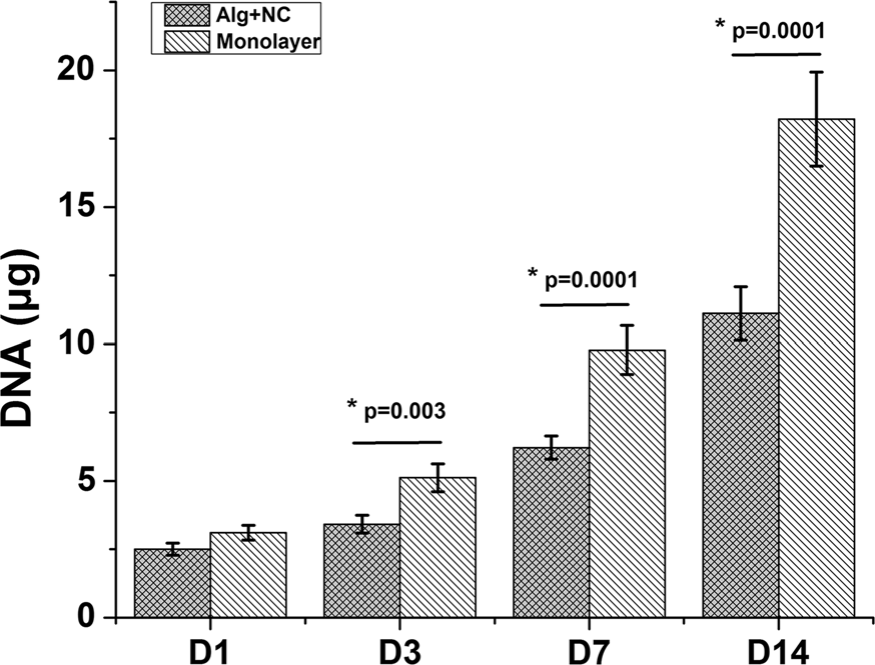
The proliferation curve of NCs cultured in 3D alginate hydrogel and monolatyer demonstrated by DNA quantification. The results are the mean ± SD (n = 4)

**Fig. 2:**
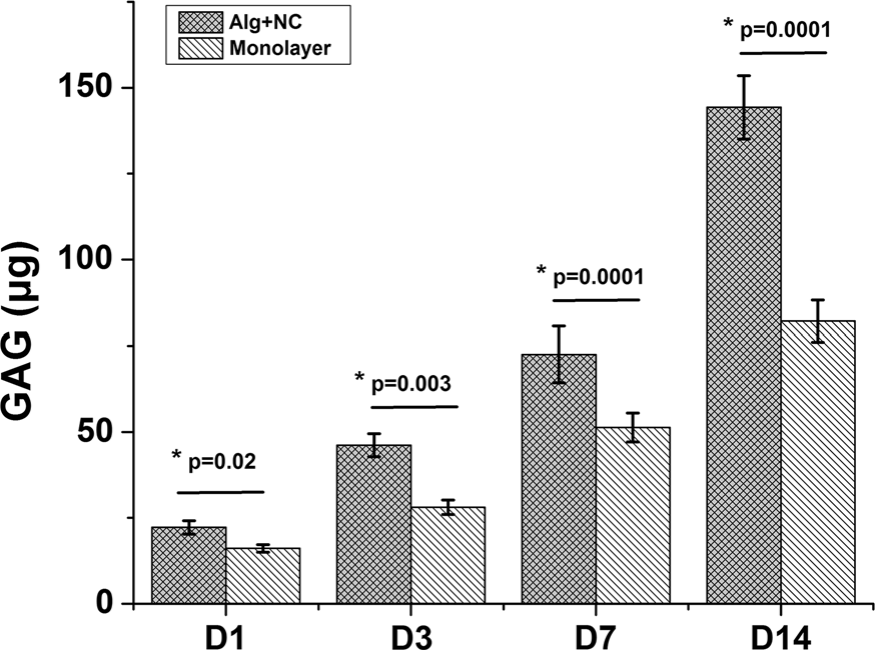
GAG quantification in 3D alginate hydrogel and monolatyer. The results are the mean ± SD (n = 4)

Cell proliferation and cartilage matrix production were demonstrated by DNA and GAG quantification. The DNA content of both 3D alginate culture group (Alg + NC group) and monolayer culture group (Monolayer group) increased during in vitro culture from day 1 to day 14. At day 1, the DNA content of Monolayer group is higher than that of Alg + NC group, but this was not statistically significant (*p* > 0.05). At day 3, 7, 14, NCs in monolayer culture proliferated significantly more than that in 3D alginate culture (*p* < 0.01) (Fig. 1). GAG is one of main components of cartilage extracellular matrix (ECM). Increase in GAG accumulation both in Alg + NC group and Monolayer group over culture time showed continuous cartilage ECM deposition. However, the GAG content of Alg + NC group was significantly more than that in Monolayer group (*p* < 0.05) (Fig. 2).

### Chondrogenic gene expression level of NCs in 3D alginate culture

**Fig. 3:**
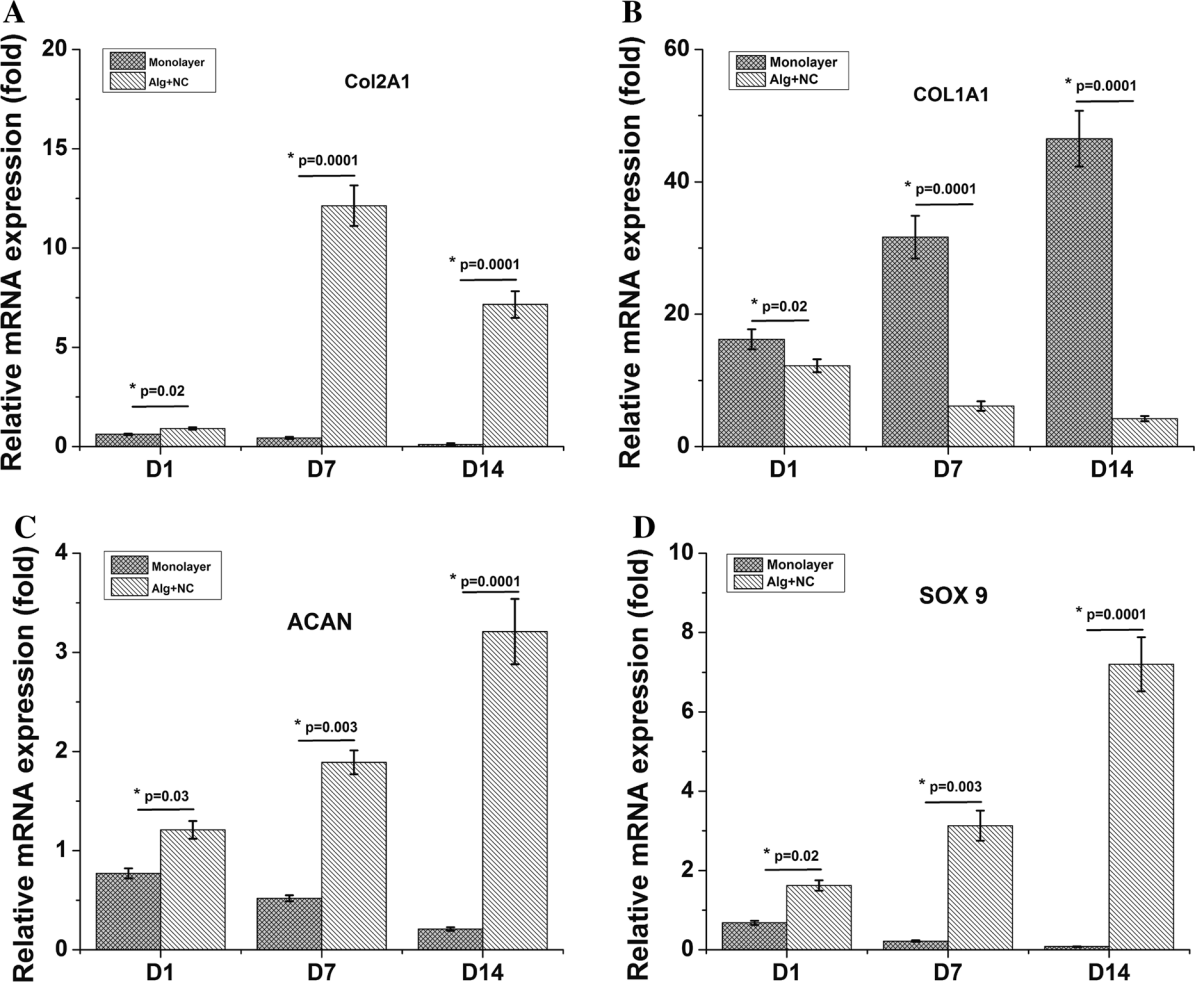
Chondrogenic gene expression level of NCs in 3D alginate culture. The results are the mean ± SD (n = 3)

QPCR was conducted to determine the mRNA expression of several chondrogenic markers (Col2A1, ACAN, SOX9) at different time point (day 1, 7, 14). Col2A1, ACAN, and SOX9 gene expression in the Monolayer group down-regulated as culture time increased. While with the extending of 3D alginate culture of NCs, these chondrogenic genes showed up-regulated continuously from day 1 to day 14, and showed higher expression levels than the Monolayer group at all the time points (Fig. 3a, c, d). Col1A1, which is regarded as a dedifferentiation marker, showed up-regulated in the Monolayer group, and down-regulated in the Alg + NC group along with the prolongation of in vitro culture time (Fig. 3b).

### Histological evaluation of repaired tissue in vivo

**Fig. 4:**
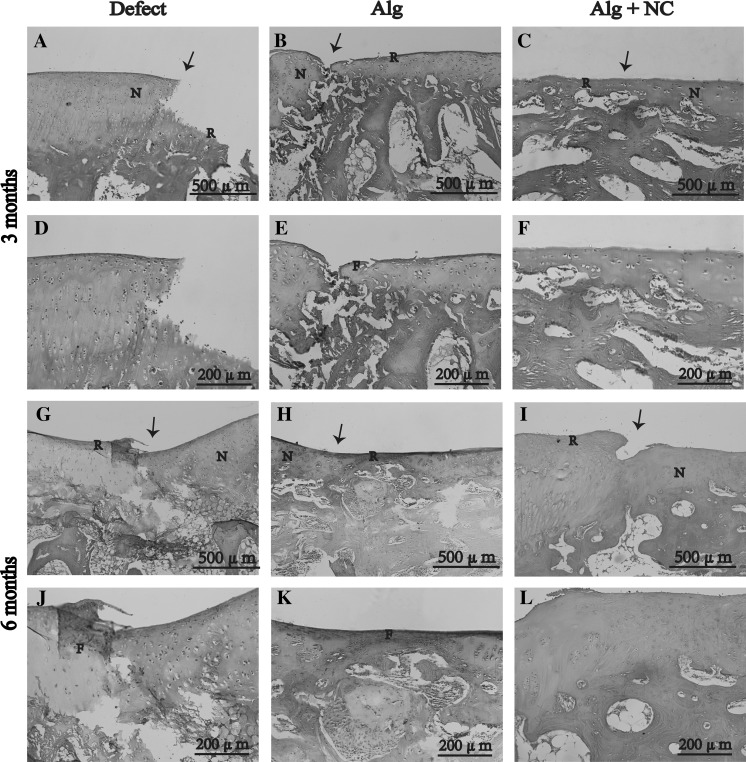
Hematoxylin and eosin staining of repaired tissue in three groups at 3 and 6 months after surgery. *N* native cartilage, *R* repaired tissue, *F* fibrous tissue. The *black arrow* indicate the margins of native cartilage and repaired tissue. **d**–**f** Are the higher magnification of repaired areas in **a**–**c**; **j**–**l** are the higher magnification of repaired areas in **g**–**i**

**Fig. 5:**
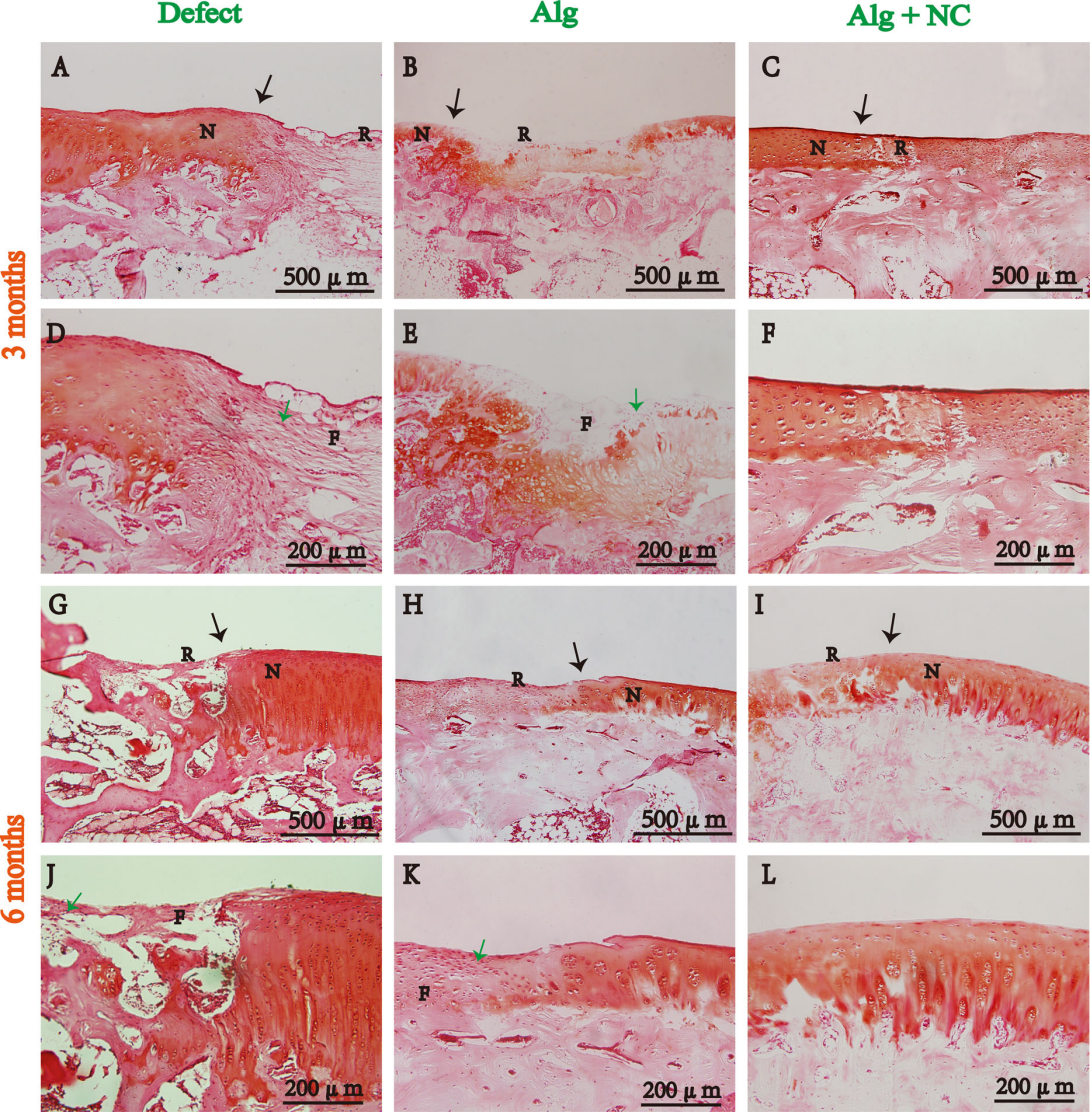
Safranin O staining of repaired tissue in three groups at 3 and 6 months after surgery. *N* native cartilage, *R* repaired tissue, *F* fibrous tissue. The *black arrow* indicate the margins of native cartilage and repaired tissue, the *green arrow* indicate the firoblast. **d**–**f** Are the higher magnification of repaired areas in **a**–**c**; **j**–**l** are the higher magnification of repaired areas in **g**–**i**. (Color figure online)

H&E staining, safranin O staining, and histological were performed for histological evaluation of repaired tissue. At 3 months after surgery, the defect in the Alg + NC group was almost filled with repaired tissue (Fig. 4c, f). In contrast, the defects in the Alg group and the Defect group were partly filled with repaired tissue with distinct boundary between the neotissue and the surrounding native cartilage (Fig. 4a, b, d, e). H&E staining showed that the repaired tissue in the Alg + NC group was mostly hyaline-like cartilage containing many chondrocytes, while the repaired tissue in the Alg group and the Defect group were mostly fibrous tissue containing many spindle-shaped fibroblasts (Fig. 4a–f). Safranin O staining showed that the repaired tissue in the Alg + NC group was strongly stained with sfranin O, which was similar to the nearby native cartilage tissue, however, the repaired tissue in the Alg group and the Defect group were rarely stained with sfranin O, which indicated poor cartilage ECM deposition (Fig. 5a–f).

At 6 months after surgery, the defect in the Alg + NC group was completely filled with repaired tissue, and the defects in the two other groups were mostly filled with neotissue. H&E staining showed that the morphology of the repaired tissue in the Alg + NC group was very similar to the surrounding native cartilage. Many chondrocytes with typical lacuna can be seen inside the repaired tissue with relatively regular arrangement (Fig. 4i, l). The repaired tissue in the Alg group was a mixture of fibrous tissue and hyaline cartilage, while the repaired tissue in the Defect group was still fibrous tissue (Fig. 4g, h, j, k). Safranin O staining showed that the repaired tissue in the Alg + NC group was intensely stained with safranin O, which was comparable to the normal cartilage tissue, indicating superior cartilage repair (Fig. 5i, l). The repaired tissue in the Defect group was hardly stained by safranin O, while light staining was observed in the Alg group (Fig. 5g, h, j, k).

Histological score ranged from 0 to 12 points. Higher scores indicate better repairs. The score of the Alg + NC group was higher than those of the Alg group and the Defect group (*p* < 0.01) (Fig. 7a).

### Micro-CT analysis of subchondral bon reconstruction

**Fig. 6:**
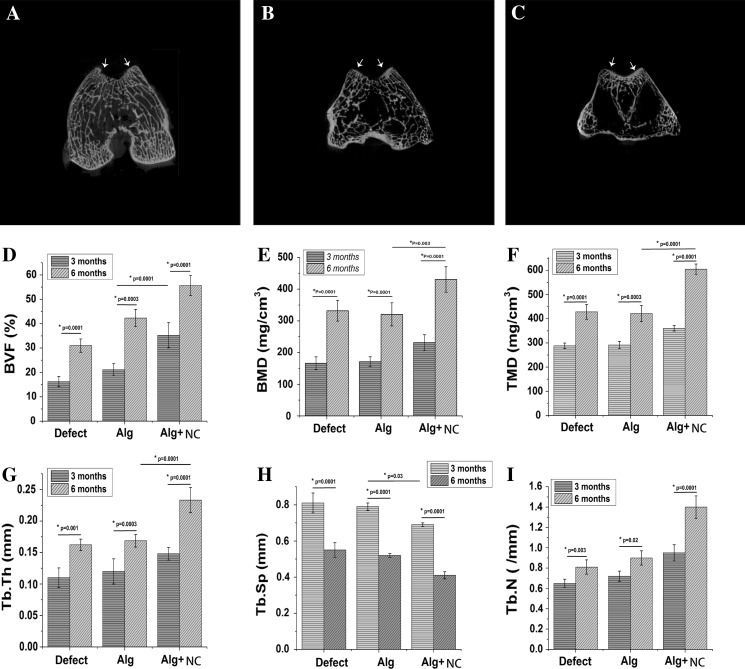
Micro-CT imaging and analysis at 3 and 6 months after surgery. **a**–**c** 2-D micro-CT images of repaired knee at 6 months after surgery. **d**-**i** Quantitative analysis of bone volume fraction (BVF), bone mineral density (BMD), tissue mineralized density (TMD), trabecular bone thickness (Tb.Th), spacing (Tb.Sp), and number of trabecular bone (Tb.N) were quantified at 3 and 6 months after surgery. The white arrows indicate the margins of the defect region. The results are the mean ± SD (n = 4)

Micro-CT analysis was conducted at 6 months after surgery to evaluate the subchondral bone reconstruction. A certain amount of new osseous tissue developed from the outer area of the subchondral bone defect was observed in all the groups, however, the regenerated bone volume in the Alg + NC group was greater than that in the Alg group and the Defect group (Fig. 6a–c). The BVF, BMD, and TMD values of the Alg + NC group was higher than the two other groups both at 3 and 6 months (*p* < 0.01) (Fig. 6d–f). The Tb.Sp value of the Alg + NC group was lower than the two other groups both at 3 and 6 months (*p* < 0.01) (Fig. 6h). With respect to the Tb.Th and Tb.n values, there was no significant difference among the three groups at 3 months, while the Alg + NC group was significantly higher than the two other groups at 6 months (Fig. 6g, i). There was no significant difference between the Alg group and the Defect group at both time points (*p* > 0.05). For each specific group, there was a significant difference between 3 and 6 months (*p* < 0.01).

### Biomechanical properties of repaired tissue in vivo

**Fig. 7:**
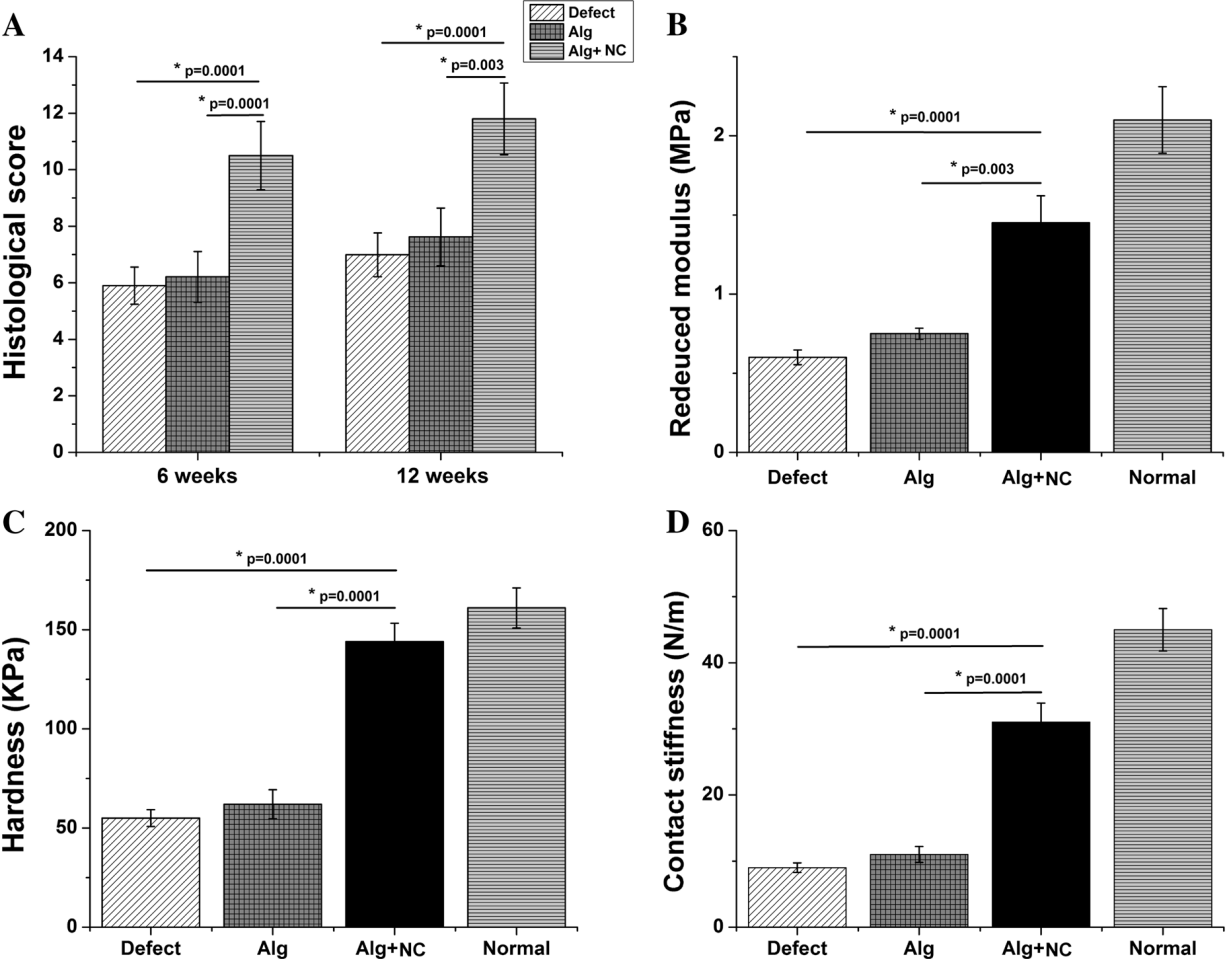
Histological scoring and biomechanical evaluation. **a** Histological scores of three groups at 3 and 6 months after surgery. **b**–**d** Reduced modulus, hardness contact stiffness of repaired tissue and normal cartilage at 6 months after surgery. The results are the mean ± SD (n = 3)

Biomechanical test was performed at 6 months after surgery to assess the mechanical properties of repaired tissue in the three groups. The reduced modulus, together with hardness and contact stiffness are considered as crucial parameters to evaluate biomechanical properties of cartilage tissue via nanoindentation testing. In our study, normal cartilage tissue showed highest reduced modulus, as well as hardness and contact stiffness. The mechanical properties of repaired tissue in the Alg + NC group were more similar to the normal cartilage tissue, and superior than those in the Alg group and the Defect group. There was no significantly difference between the Alg group and the Defect group (*p* > 0.05) (Fig. 7b–d).

## Discussion

Cartilage repair remains elusive due to its very limited ability of natural healing and poor response to treatment (Huey et al. 2012). Tissue engineering strategy has been applied to induce cartilage regeneration. The seed cell source is one of the key factors to the success of cartilage tissue engineering approaches for cartilage repair. In clinic, autolougous ACs are regarded as one of the best cell sources. For this strategy, patients have to undergo two operations. The first operation is conducted to harvest cartilage biopsy from the nonweight bearing areas of the joint. After in vitro expansion, autologous chondrocytes combined with bioscaffold or not are re-transplanted to the cartilage injury site of the same patient by second operation. Whereas, several drawbacks such as twice surgical trauma, donor site morbidity, chondrogenic phenotype loss during in vitro chondrocytes expansion may lead to inferior cartilage repair (Matsunaga et al. 2007; Foldager 2013; Hinckel and Gomoll 2017). Considering these limitations, in our study, we were trying to identify the potential of NCs for in vivo articular cartilage repair.

NCs and ACs harbor in tissues with a common characteristic of hyaline cartilage and secrete similar ECM components. However, NCs and ACs develop from different germinal layers. NCs arise from the neuroectoderm, while ACs from mesoderm (Pelttari et al. 2014). Studies have revealed that cells origin from neuroectoderm showed higher self-renewal capacity and greater regenerative ability than those from mesoderm associated with HOX gene expression (HOX negative for neuroectoderm origin, positive for mesoderm origin) (Grapin-Botton et al. 1995; Couly et al. 1998). W. Kafienah et al. (2002) reported that human NCs exhibit greater chondrogenic potential and higher capacity to generate hyaline cartilage tissue than ACs. Candrian et al. (2008) reported that engineered cartilage by NCs showed to response to physical forces resembling joint loading, which is very similar to ACs. When exposed to inflammatory factors interleukin-1β and low oxygen mimicking the post-surgery articular environment, human NCs could recover efficiently, similar or even more better than ACs (Scotti et al. 2012). What’s more, NCs are very easy to harvest from a small nasal septum biopsy, with minimal donor site morbidity. Collectively, these findings suggest that NCs could represent a very promising cell source for articular cartilage repair.

Biomaterials, which can act as cell carriers and provide structural support, and even influence cell biological manners, are also vital for cartilage tissue engineering. Hydrogel, especially naturally derived hydrogel have been extensively studied for the following advantages: (1) excellent biocompatibility; (2) easier handling than hard scaffold; (3) can be injected in vivo with very minimally invasion; (4) complete defect filling; (5) reach very deep tissue defect (Maisani et al. 2017; Yao et al. 2017). Alginate, with a tunable and glycosaminoglycans like structure, has already been explored in many studies as injectable cell delivery vehicle for a very wide range of applications. In the field of cartilage regeneration, alginate can provide a cartilage ECM-mimicking microenvironment for chondrocytes proliferation and maintain their chondrogenic phenotype during in vitro culture (Sharma et al. 2016; Silva et al. 2016). In our study, rabbit NCs were firstly cultured in 3D alginate hydrogel. The results showed that NCs can proliferate stable in 3D alginate matrix as revealed by increased DNA contents with culture time prolonged. The proliferation rate of NCs cultured in monolayer was faster than that cultured in 3D alginate hydrogel (Fig. 1). However, the GAG content of NCs cultured in 3D alginate was significantly more than that in monolayer group (Fig. 2). QPCR results revealed that NCs expressed chondrogenic markers (Col2A1, ACAN SOX9). With culture in monolayer, Col2A1, ACAN, and SOX9 genes expression were down regulated, while Col1A1 gene was up regulated, indicating NCs undergo the dedifferentiation process in 2D monolayer culture, which is in consistent with ACs. Three-dimensional culture of NCs within alginate was shown to maintain the expression of chondrogenic markers (Col2A1, ACAN SOX9) and down regulate the expression of dedifferentiation marker (Col1A1) (Fig. 3). These results emphasize that alginate can support NCs proliferation and maintain its chondrogenic phenotype and the ability of producing cartilage extracellular matrix continuously. Therefore, these findings suggest that alginate can serve as suitable delivery carrier of NCS for cartilage tissue engineering.

Further, we explored the use of injectable NCs and alginate hybrid construct for in vivo articular cartilage repair in a rabbit model. Hyaline cartilage repair was induced in the Alg + NC group as revealed by histological assessment, while the groups treated with alginate alone or left empty were mostly repaired by fibrous tissue. Although NCs and ACs derive from different germinal layers, the organization of the regenerated hyaline cartilage which induced by NCs are very similar to that of the surrounding native articular cartilage. This may associated with the adaptation of a HOX-positive profile of NCs upon implantation into a knee joint, which recognized as mesoderm environment, which has already been reported by Pelttari et al. (2014). Micro-CT data indicated that the Alg + NC group obtained better subchondral bone reconstruction than the Alg group and the Defect group. Nanoindentation testing indicated that the biomechanical properties of repaired tissue in the ALG + NC group are very similar to native cartilage.

## Conclusions

Our study demonstrated that autologous NCs can induce superior articular cartilage regeneration in a rabbit osteochondral defect model. These results therefore suggest autologous NCs are very promising cell source for articular cartilage repair and highlight the potential of alginate hydrogel as injectable cell delivery system for cell-based therapy of articular cartilage injury with very minimally invasion.
